# P02 When should we stop the antiresorptive medications in patients with osteoporosis?

**DOI:** 10.1093/rap/rkad070.023

**Published:** 2023-09-27

**Authors:** Marwa Mohareb, Anupama Nandagudi

**Affiliations:** Basildon, Basildon, United Kingdom; Basildon, Basildon, United Kingdom

## Abstract

**Introduction:**

Bisphosphonates are first line and denosumab is the latest antiresorptive agent approved for osteoporosis treatment. Those drugs showed a reduction in vertebral fracture risk and other non-vertebral by 35-70%.

One of the rare adverse events with antiresorptive treatment is medicine-related osteonecrosis of jaw (MONJ) which mainly occur in individuals receiving high doses for the treatment of cancer.

Recent research found true incidence of MONJ is very low despite public perception. Studies indicate that 50% of women and 20% of men aged over 50 years will experience an osteoporosis-related fracture which have significant associated morbidity and mortality.

**Case description:**

We report a case of an osteoporosis patient who was treated with alendronate followed by denosumab and presented with suspicion of MONJ.

In 2014, a 77-year-old female was referred following right neck of fragility femur fracture. DXA showed osteoporosis (T- score of the spine −3.2, T- score total hip −2.4 and T- score of left neck of femur −2.1). She was initially treated with four years of alendronate (70 mg once weekly) but sustained a pubic rami fracture. Hence, she was then treated with six monthly injections of denosumab (60 mg subcutaneously).

After three injections, she developed jaw pain. She was reviewed by her dentist who advised her to stop the denosumab for suspected MONJ. She was then seen by the oral surgeon who confirmed that there was a piece of bone in UR6 socket but with no bone sequestrum and this piece of bone was removed under local anaesthesia with good healing. She was advised to restart denosumab.

The patient was extremely worried and anxious about denosumab recommencement and teriparatide was considered.

After completion of teriparatide treatment her DXA still showed osteoporosis (T- score of the spine −2.1, T- score of total hip −2.2 and T- score of left neck of femur −2.6) with stable changes in the hip and improvement in the spine. On review, she continued to have concerns about antiresorptive treatment.

Further oral surgeon review stated low grade infection around UL7 with poor gum condition around UR6 and LR6 with some bleeding points but no definite diagnosis of MRONJ. It was advised that any further manoeuvre involving tooth extraction will be conducted by the oral surgeon.

Following this, the patient was happy to consider zoledronic acid infusions. She is due for her 3rd zoledronate infusion and is undergoing regular dental checks.

**Discussion:**

The incidence of MONJ in patients taking oral bisphosphonates for osteoporosis is relatively low, estimated to be less than 1% (0.001% to 0.01%). However, the risk is higher for individuals receiving higher doses of intravenous bisphosphonates for the treatment of cancer or bone-related complications of cancer. The incidence of MONJ in cancer patients receiving intravenous bisphosphonates ranges from 1% to 15%.

More clinical studies have been published that describe the relation between MONJ and denosumab in cancer patients with the risk of MONJ varying between 0.7 to 1.9% (70 to 90 cases per 10,000 patients). However, in patients with osteoporosis treated with denosumab, the incidence of MONJ is lower, resulting in an even lower frequency of 0.04% (4 cases per 10,000 patients).

It is understandable that there is a significant concern amongst dentists about MONJ risk with antiresorptive treatment, but it is paramount that the patients are made aware of the fracture risk and its significant effect on quality of life. Many patients are not aware that more than 20% of patients die in the first year after a hip fracture from complications surrounding the fracture and a significant number of patients lose their long-term ability to live independently. Vertebral fractures have significant morbidity and the mortality rate of 72% at 5 years and 90% at 7 years.

**Key learning points:**

Most dentists are often reluctant to treat patients on anti-resorptive medications due to the risk of MONJ which results in patient’s concerns about treatment and subsequent stoppage of osteoporosis treatment leading to fracture risks. Thus, it is imperative to assure the patients of benefits of the anti-resorptive medications versus the MONJ risks. This calls for increased awareness and national guidelines for the prevention and management of ONJ in osteoporosis patients treated with antiresorptive.

